# Hyperdense middle cerebral artery sign is a prognostic factor of favourable long-term outcomes of mechanical thrombectomy in acute ischaemic stroke

**DOI:** 10.1007/s00234-025-03871-z

**Published:** 2025-12-27

**Authors:** Pawel Wrona, Mateusz Gielczynski, Aleksandra Wojnarska, Katarzyna Sawczynska, Helin Savsin, Katarzyna Chwaleba, Tomasz Homa, Roman Pulyk, Agnieszka Slowik

**Affiliations:** 1 Department of Neurology, Jagiellonian University Medical College, Krakow, Poland; 2 Department of Neurology, University Hospital in Krakow, Krakow, Poland; 3 Department of Radiology, University Hospital in Krakow, Krakow, Poland; 4 Student Scientific Group in Cerebrovascular Diseases, Faculty of Medicine, Jagiellonian University Medical College, Krakow, Poland

**Keywords:** Acute ischaemic stroke, Hyperdense middle cerebral artery sign, Mechanical thrombectomy, Functional outcome.

## Abstract

**Purpose:**

Hyperdense middle cerebral artery sign (HMCAS) is a phenomenon highly specific for acute ischaemic stroke (AIS) that can be found in brain non-contrast computed tomography (NCCT). Previous studies concerning its association with outcomes of patients undergoing mechanical thrombectomy (MT) are inconclusive. Our aim was to assess the relationship between HMCAS presence and long-term outcomes of AIS patients undergoing MT.

**Methods:**

The study included anterior circulation AIS patients treated with MT in the University Hospital in [ANONYMIZED] from 2019 to 2021, in whom admission NCCT and one-year follow-up were available. The clinical, laboratory and imaging data, as well as following outcomes: the occurrence of successful recanalization [defined as modified treatment in cerebral infarction (mTICI) score 2b-3], haemorrhagic complications (ICH), 90-day and 365-day rates of mortality and good functional outcome [defined as modified Rankin Scale (mRS) score 0–2] were compared between groups of patients with and without HMCAS on initial NCCT. The association of HMCAS presence with the abovementioned outcomes was assessed using multivariate logistic regression analysis.

**Results:**

Among 359 MT-treated patients with anterior circulation AIS, HMCAS was found in 244 (67.97%). The presence of HMCAS was independently associated with good functional outcome at 365 days (OR 1.956, 95% CI = 1.152–3.317, *p* = 0.013) as well as lower 90-day and 365-day mortality (OR = 0.464, 95% CI = 0.2517–0.856; *p* = 0.014 and OR 0.543, 95% CI: 0.313–0.940, *p* = 0.029, respectively).

**Conclusion:**

The presence HMCAS on admission NCCT is associated with favourable long-term outcome in AIS patients undergoing MT.

## Introduction

Hyperdense middle cerebral artery sign (HMCAS) is seen in brain non-contrast computed tomography (NCCT) as unilateral, increased density of the middle cerebral artery (MCA), higher than the surrounding tissues and disappearing on bone window [1, 2]. It can be found in 20–36% of anterior circulation acute ischaemic stroke (AIS) patients [3, 4]. It is a highly specific (94–95%) indicator of the presence of large vessel thrombus in the early stage of AIS [4, 5].

The association of this radiological finding with poor functional outcomes was proven in patients with AIS undergoing intravenous thrombolysis [6]. However, in occlusions of large vessels (including MCA), causative AIS treatment includes also mechanical thrombectomy (MT) [7], in which case the correlation of HMCAS presence with treatment outcomes is less defined. Current studies on this matter produce mixed results [8–15]. To the best of our knowledge, no studies have yet assessed outcomes of HMCAS (+) AIS patients undergoing MT in observations longer than 90 days.

Identification of prognostic factors in AIS patients undergoing MT is important for risk stratification and patient counselling. HMCAS evaluation is easy to assess and available in all patients undergoing NCCT, so potentially it could be a useful tool for outcome prediction. Therefore, the aim of this study is to determine the association of HMCAS presence with long-term (365-day) outcomes of MT.

## Materials and methods

**Fig. 1:**
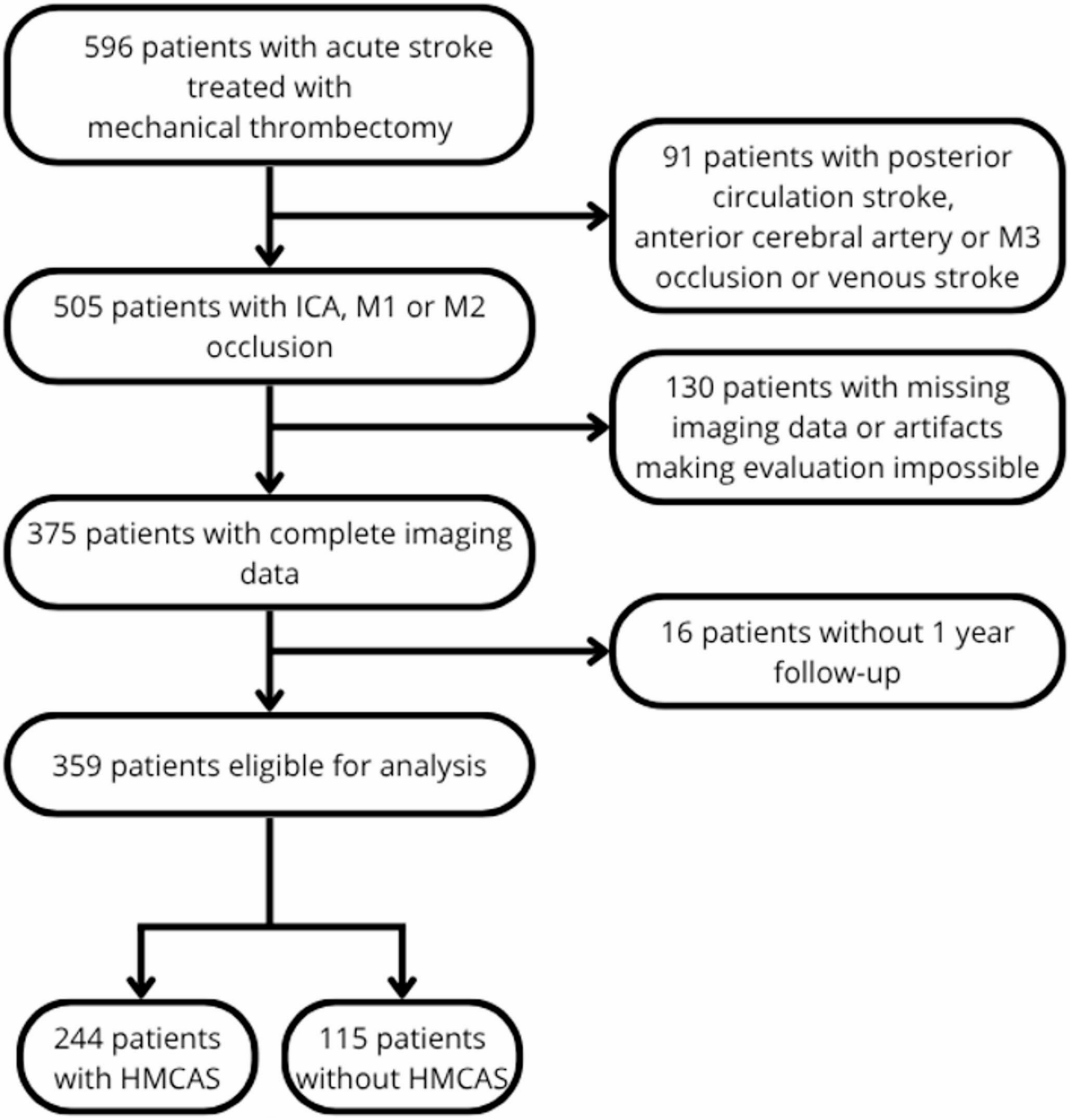
Study flow-chart showing the inclusion and exclusion of the patients

We conducted a retrospective analysis on data that was prospectively collected for the purpose of iBioStroke study (Identification and clinical validation of biomarkers for long-term outcome after cerebral ischaemia, Jagiellonian Bioethics Committee approval number KBET/1072.6120.118.2020) including all AIS patients hospitalised in the Comprehensive Stroke Centre of the University Hospital in Krakow from January 2019 to December 2021. AIS diagnosis was consistent with the AHA/ASA definition [16]. For the purpose of the presented study, we analysed data from the subpopulation of patients with AIS and anterior circulation large vessel occlusion (LVO) involving the internal carotid artery (ICA), middle cerebral artery (MCA) M1 or M2 segment who were treated with mechanical thrombectomy (MT). This included patients with T-occlusion, defined as occlusion of terminal portion of ICA involving initial parts of MCA and anterior cerebral artery (ACA) [17, 18]. Inclusion criteria and the percentages of exclusions due to various reasons are presented in Fig. 1.

### Clinical assessment

In all patients we collected the following data: demographics (age and sex), stroke risk factors profile: (1) arterial hypertension (diagnosed in previous medical history and/or antihypertensive treatment prior to stroke onset and/or systolic blood pressure ≥ 140 mmHg or diastolic blood pressure ≥ 90 mmHg at least in two different measurements after the first three days of hospitalisation); (2) atrial fibrillation (diagnosed in previous medical history or during hospitalisation based on electrocardiograms); (3) history of myocardial infarction in patient’s medical history; (4) carotid artery atherosclerosis (intima-media complex thickening or presence of atherosclerotic plaques, with stenoses >50% considered haemodynamically significant); (5) stroke or transient ischaemic attack (TIA) in previous medical history; (6) diabetes or prediabetes (diagnosed according to ESC criteria [7]); (7) dyslipidaemia (defined as a cholesterol level >5.2 mmol/L or use of cholesterol–lowering treatment); (8) peripheral artery disease (presence of atherosclerotic plaques in arteries other than coronary and cerebral confirmed by ultrasound during hospitalisation or in previous medical history); (9) history of smoking during the last 15 years; (10) obesity (BMI >30 kg/m²), 11. history of cancer (diagnosis of malignancy within 5 years preceding the onset of AIS and undergoing cancer-specific treatment during this time or being in the process of qualification for it). We evaluated the patients for previous antithrombotic treatment history (regular use of antiplatelet and anticoagulant medicines in previous three months). Clinical assessment also included: stroke severity on admission assessed with the National Institutes of Health Stroke Scale (NIHSS), prevalence of pneumonia and urinary tract infections, blood test results (first ones available after admission) including serum glucose level, blood cell count (WBC), haemoglobin levels (Hb), platelet count (PLT), creatinine, fibrinogen, activated partial thromboplastin time (APTT), international normalized ratio (INR), C-reactive protein (CRP). Stroke aetiology was classified according to the Trial of ORG 10,172 in Acute Stroke Treatment (TOAST) criteria [19].

### Treatment

Comprehensive Stroke Center at the University Hospital in [ANONYMIZED] provides MT for a population of 3.36 million inhabitants. AIS patients are admitted to the nearest stroke unit (15-unit Network) in the region or the CSC, if indicated, where intravenous thrombolysis (IVT) is performed. Those fulfilling the MT criteria are admitted to the CSC. All patients were treated according to the selection and eligibility criteria of the current European Stroke Organisation (ESO) guidelines on intravenous thrombolysis and mechanical thrombectomy in AIS [7, 20], therefore inclusion criteria for MT were: LVO-related acute ischaemic stroke presenting within 6 h after symptom onset or LVO-related acute ischaemic stroke presenting between 6 and 24 h from time last known well and fulfilling the perfusion volumetry criteria equivalent to DEFUSE-3 or DAWN trials [21, 22]. The minimum age threshold was 18 years, with no upper age limit for patients treated within the 6-hour window. Age thresholds for the extended time windows followed the relevant protocols mentioned above. Major exclusion criteria included: intracranial hemorrhage on NCCT, recent head trauma, pregnancy, known allergy to iodine contrast, severe organ failure, life expectancy less than 6 months, uncontrolled hypertension (systolic ≥ 185 or diastolic ≥ 110 mmHg), blood glucose level of < 50 mg/dL or >400 mg/dL and thrombocytopenia (< 50 × 10⁹/L).

### Neuroimaging

Patients with AIS qualified for MT underwent the following radiological protocol: initial non-contrast computed tomography (NCCT), initial computed tomography angiography (CTA), initial computed tomography perfusion (CTP), post-processing analysis with RAPID software (iSchema view, Menlo Park, CA, USA) including eASPECT score, infarct volume defined as cerebral blood flow (CBF) less than 30% volume, penumbra volume defined as volume of tissue with Time to maximum (Tmax) of more than 6 s minus infarct volume. Follow-up NCCT was performed 24 h after MT.

All non-contrast CT (NCCT) that were performed in the Comprehensive Stroke Center were done using Siemens Somatom Definition Edge 64-detector scanner before the endovascular procedure. NCCT scans were reviewed by experienced radiologist and neurologist separately without access to results of recanalization procedure. Hyperdense middle cerebral artery sign (HMCAS) was defined as unilateral, increased density of the MCA, higher than surrounding tissues, disappearing on bone window and in absence of subarachnoid hemorrhage [8, 38]. If there were discrepancies in the assessment of HMCAS, a consensus was made between specialists.

### Thrombectomy procedure

We recorded the following concerning MT procedure: site of occlusion, recanalization as measured the modified treatment in cerebral infarction (mTICI) score, time lapses that have an impact on stroke outcomes: time from stroke onset to groin puncture (SO-GP), time from groin puncture to recanalization (GP-RT), time from door-to-needle (DTN). All mechanical thrombectomy procedures were performed by experienced interventional radiologists. The thrombectomy method—aspiration, stent retriever, or a combination of both—was determined at the operator’s discretion based on angiographic visualization of the clot, with first-pass aspiration used as the initial technique when technically feasible. In cases of severe stenosis, balloon angioplasty and stent implantation were also performed.

### Outcome measures

The following outcomes were assessed:

1. presence of any intracerebral hemorrhage (ICH) on NCCT performed 24 h after MT,
2. successful recanalization after MT procedure defined as mTICI score of 2b or 3,
3. in-hospital mortality,
4. 90-day good functional outcome, defined as modified Rankin Scale (mRS) score 0–2,
5. 90-day mortality,
6. 365-day good functional outcome, defined as mRS score 0–2
7. 365-day mortality.

Follow-up functional outcome was evaluated during scheduled visits in the outpatient clinic of our Centre or through telephone interviews with patients or their family members/caregivers.

### Statistical analysis

Statistical analysis was conducted using the PS Imago Pro 9.0 program (Predictive Solutions, Kraków, Poland). The data was presented in two forms: categorical data was expressed as counts and percentages, while continuous data was presented as mean and 95% confidence interval (CI) or median and interquartile range (IQR) depending on normality, which was tested with Kolmogorov-Smirnov test. Categorical data was compared between groups of HMCAS (+) and HMCAS (-) patients using the Chi-square test. For normally distributed continuous data, the t-Student test was used to compare between groups; for non-normally distributed data, the Mann-Whitney U test was used. The threshold for statistical significance was set at a two-sided p-value of less than 0.05.

Statistically significant variables identified from the previous analysis were used to perform logistic regression for each outcome. Clinically relevant variables that did not meet statistical significance were also included where appropriate. Univariate logistic regression analyses were first conducted to evaluate the individual contribution of each variable to the outcomes. Selected factors were then assessed for statistical significance (*p* < 0.1) and clinical relevance and included in multivariate analysis models if fullfiling any of these criteria. To assess multicollinearity, the Variance Inflation Factor (VIF) was calculated for each variable, with a maximum acceptable threshold of 5. The models were evaluated with Hosmer-Lemeshow test for goodness of fit. Models with *p* > 0.05 were used to calculate the odds ratios (OR) and 95% confidence intervals describing influence of variables on the outcomes.

### Ethics and funding

All procedures were performed in accordance with the Declaration of Helsinki. Written consent was obtained from all patients before the start of any research procedures. The study was approved by the Jagiellonian University Ethical Committee (KBET 1072.6120.118.2020, 1072.6120.256.2022).

## Results

### Study population

Between January 2019 and December 2021 there were 505 patients with anterior circulation AIS treated with MT in the Comprehensive Stroke Center in [ANONYMIZED]. Out of those, 359 had complete imaging data suitable for the analysis of HMCAS presence and completed one-year follow up observation. Detailed inclusion data is presented in Fig. 1.

The included patients were aged 23 to 97 years with a median age of 71 (IQR 63–81) and 179 (49.86%) were female. In 244 (67.97%) HMCAS was found in initial NCCT.

### Comparison of HMCAS (+) and (-) patients

HMCAS (+) patients did not differ significantly from HMCAS (-) patients in terms of age, sex and occurrence of cardiovascular risk factors with the exception of less common history of cancer (4.91% vs. 11.30%, *p* = 0.027). They differed in the occurrence of particular artery occlusions: ICA (13.52% vs. 29.57%, *p* < 0.001), M1 (61.07% vs. 48.70%, *p* = 0.027) and T-occlusion (6.97% vs. 0.87%, *p* = 0.017). They were more commonly treated with IVT (62.30% vs. 50.43%, *p* = 0.033) and had higher penumbra volume on admission [median of 101 ml (IQR 67–131.5.5 ml**)** vs. 70 ml (IQR 30.5–114 ml), *p* < 0.001)]. There were no differences between the groups concerning stroke aetiology (according to TOAST classification), prevalence of infections requiring antibiotic therapy, blood sample results, antithrombotic therapy or analysed time lapses (SO-GP, GP-RT, DTN).

Detailed group comparison concerning baseline characteristics of the patients is presented in Table 1.

HMCAS patients had lower long-term mortality, both 90-days (18.44% vs. 29.57%, *p* = 0.018) and 365-days (26.23% vs. 37.39%, *p* = 0.031) after the procedure. We also observed higher percentage of good functional outcomes after 365 days in HMCAS (+) group (65.16% vs. 52.17%, *p* = 0.019). We found no differences between the groups in the occurrence of ICH, good recanalization rates, in-hospital mortality and 90-day good functional outcome.

Outcomes are presented in detail in Table 2.

**Table 1:** Baseline characteristics of the patient groups with and without HMCAS

	HMCAS (*n* = 244)	No HMCAS (*n* = 115)	*p*-value
Demographic data			
Age [years], median (IQR)	71 (64–81)	70 (63–80)	0.371
Female sex, *n* (%)	120 (49.18)	59 (51.3)	0.707
Comorbidities			
Arterial hypertension, *n* (%)	176 (72.13)	84 (73.04)	0.857
Atrial fibrillation, *n* (%)	114 (46.72)	45 (39.13)	0.177
Diabetes mellitus, *n* (%)	51 (20.9)	28 (24.35)	0.462
Hypercholesterolemia, *n* (%)	54 (22.13)	22 (19.13)	0.516
Carotid artery atherosclerosis, *n* (%)	50 (20.49)	28 (24.35)	0.408
Peripheral artery disease, *n* (%)	20 (8.20)	11 (9.57)	0.667
History of cancer, *n* (%)	12 (4.91)	13 (11.30)	0.027
Obesity, *n* (%)	52 (21.31)	18 (15.65)	0.207
History of smoking, *n* (%)	50 (20.49)	28 (24.35)	0.420
History of myocardial infarction, *n* (%)	32 (13.11)	16 (13.91)	0.836
History of stroke/TIA, *n* (%)	31 (12.7)	13 (11.3)	0.706
Prior antithrombotic treatment			
Use of antiplatelet agents, *n* (%)	58 (23.77)	28 (24.35)	0.937
Use of anticoagulants, *n* (%)	57 (23.36)	31 (26.96)	0.486
Location of occlusion			
ICA occlusion, *n* (%)	33 (13.52)	34 (29.57)	< 0.001
M1 occlusion, *n* (%)	149 (61.07)	56 (48.70)	0.027
M2 occlusion, *n* (%)	45 (18.44)	24 (20.87)	0.586
T-occlusion, *n* (%)	17 (6.97)	1 (0.87)	0.017
Baseline clinical and radiological characteristics and information on reperfusion therapy			
NIHSS score at admission, median (IQR)	16 (10–20)	15 (9–19)	0.212
ASPECTS, median (IQR)	8 (7–9)	9 (7–10)	0.116
Intravenous thrombolysis, *n (*%)	152 (62.3)	58 (50.43)	0.033
Door to needle time [min], median (IQR), *n* = 210	35 (22–50)	39.5 (23.75–60.25)	0.459
Time from onset to groin puncture [min], median (IQR)	270 (193.25–355)	289 (216–345)	0.314
Duration of MT [min], median (IQR)	59 (40–80)	60 (45–90)	0.294
Drip and ship model, *n* (%)	135 (55.33)	67 (58.26)	0.601
Perfusion data (*n* = 344)			
Penumbra volume at admission [ml], median (IQR)	101 (67–131.5.5)	70 (30.5–114)	< 0.001
Infarct volume at admission [ml], median (IQR)	10 (0–32.75.75)	7.5 (0–25)	0.149
Blood tests results			
Hemoglobin [g/dl], mean (95% CI)	13.06 (12.85–13.27)	12.92 (12.5–13.34.5.34)	0.282
White blood cells [x10^3/uL], median (IQR)	8.96 (7.41–11.51)	9.3 (6.92–12.65)	0.547
Platelets [x10^3/uL], median (IQR)	213 (170.5–248.5.5.5)	210 (163–271.5.5)	0.772
INR, median (IQR)	1.04 (0.98–1.12)	1.04 (0.98–1.14)	0.808
APTT [s], median (IQR)	28.1 (26.3–31)	28.7 (26.45–32.4)	0.373
Creatinine *[*µmol*/*l], median (IQR)	79.15 (65.55–96.63)	83.3 (67–101)	0.287
Glucose [mmol/l], median (IQR)	6.33 (5.1–7.71)	5.8 (4.98–8.98)	0.486
CRP [mg/l], median (IQR)	6.13 (2.81–16.15)	9 (3.6–22.95.6.95)	0.061
Fibrinogen [g/l], median (IQR)	2.7 (2.2–3.4)	2.75 (2.13–3.7)	0.510
Aetiology			
Cardioembolic, *n* (%)	111 (45.49)	44 (38.26)	0.197
Large vessels disease, *n* (%)	37 (15.16)	27 (23.48)	0.055
Rare, *n* (%)	8 (3.28)	4 (3.48)	0.922
Undetermined, *n* (%)	88 (36.06)	40 (34.78)	0.813
Other information about hospitalization			
Pneumonia, *n* (%)	56 (22.95)	36 (31.3)	0.091
Urinary tract infection, *n* (%)	38 (15.57)	25 (21.74)	0.152
NIHSS score at discharge (median, IQR)	4 (1–12)	5 (2–14)	0.320

*HMCAS* hyperdense middle cerebral artery sign, *IQR* interquartile range, *TIA* transient ischemic attack, *NIHSS* National Institute of Health Stroke Scale, *ASPECTS* Alberta stroke programme early Computed Tomography score, *MT* mechanical thrombectomy, *INR* international normalized ratio, *APTT* activated partial thromboplastin time, *CRP* C-reactive protein

**Table 2:** Comparison of outcomes of the patient groups with and without HMCAS

Outcomes			
Hemorrhagic transformation, *n* (%)	71 (29.1)	26 (22.61)	0.167
Successful recanalization (mTICI 2b-3), *n* (%)	216 (88.52)	95 (82.61)	0.124
In-hospital mortality, *n* (%)	27 (11.07)	19 (16.52)	0.140
3-months good functional outcome, *n* (%)	147 (60.25)	64 (55.65)	0.409
3-months mortality, *n* (%)	45 (18.44)	34 (29.57)	0.018
12-months good functional outcome, *n* (%)	159 (65.16)	60 (52.17)	0.019
12-months mortality, *n* (%)	64 (26.23)	43 (37.39)	0.031

*mTICI* modified treatment in cerebral infarction score

### Logistic regression analysis

During univariate logistic regression analysis HMCAS presence was found to be significantly associated with lower 90-day and 365-day mortality and better 365-day good functional outcomes. No significant relationship was found between HMCAS presence and other assessed outcomes. Based on the results of the screening analyses (Table 1), the following variables were included in the univariate logistic regression analysis (according to the criterion of p-value < 0.05): HMCAS, intravenous thrombolysis, penumbra, infarct, cancer and occlusions of: ICA, T-occlusion, and M1 (occlusions other than M2). Although TICI did not meet the inclusion criterion, it was added to the analysis due to its significant clinical relevance. Aforementioned variables, with the exception of thrombus locations (and TICI in mortality outcomes) were statistically significantly associated with outcomes in univariate analysis (*p* < 0.1). The remaining factors were also included in multivariate analysis because of clinical significance. All factors passed the multicollinearity assessment, which confirmed that each had a VIF below 2.

The results showed that HMCAS presence was associated with lower 90-day and 365-day mortality and better 365-day good functional outcomes, independent of other clinical and imaging variables. The goodness of fit for all models was confirmed by Hosmer-Lemeshow test with results noted in the appropriate headings.

Detailed multivariable analysis is presented in Table 3.

**Table 3:** Multivariate analysis of factors associated with long-term outcomes (90-day and 365-day mortality and 365-day good functional outcome) after mechanical thrombectomy

Good functional outcome at 365 days follow-up (Hosmer-Lemeshow: *p* = 0.988)			
**HMCAS**	*p* = 0.013	**OR = 1.956**	**95% CI: 1.152–3.317**
Intravenous thrombolysis	*p* = 0.043	OR = 1.656	95% CI: 1.016–2.701
Penumbra volume at admission [ml]	*p* = 0.005	OR = 0.994	95% CI: 0.991–0.998
Infarct volume at admission [ml]	*p* < 0.001	OR = 0.988	95% CI: 0.981–0.995
History of cancer	*p* = 0.207	OR = 0.547	95% CI: 0.214–1.396
ICA occlusion	*p* = 0.987	OR = 1.007	95% CI: 0.453–2.237
T-occlusion	*p* = 0.335	OR = 0.563	95% CI: 0.175–1.812
M1 occlusion	*p* = 0.496	OR = 0.800	95% CI: 0.421–1.520
Successful recanalization	*p* < 0.001	OR = 5.048	95% CI: 2.424–10.511
**Mortality at 365 days follow-up** (Hosmer-Lemeshow: *p* = 0.601)			
**HMCAS**	*p* = 0.029	**OR = 0.543**	**95% CI: 0.313–0.940**
Intravenous thrombolysis	*p* = 0.047	OR = 0.595	95% CI: 0.356–0.992
Penumbra volume at admission [ml]	*p* = 0.002	OR = 1.006	95% CI: 1.002–1.010
Infarct volume at admission [ml]	*p* = 0.001	OR = 1.010	95% CI: 1.003–1.017
History of cancer	*p* = 0.056	OR = 2.461	95% CI: 0.978–6.193
ICA occlusion	*p* = 0.672	OR = 0.834	95% CI: 0.360–1.934
T-occlusion	*p* = 0.957	OR = 1.035	95% CI: 0.294–3.650
M1 occlusion	*p* = 0.962	OR = 1.037	95% CI: 0.529–2.033
Successful recanalization	*p* < 0.001	OR = 0.250	95% CI: 0.124–0.502
**Mortality at 90 days follow-up** (Hosmer-Lemeshow: *p* = 0.805)			
**HMCAS**	*p* = 0.014	**OR = 0.464**	**95% CI: 0.251–0.858**
Intravenous thrombolysis	*p* = 0.333	OR = 0.751	95% CI: 0.421–1.340
Penumbra volume at admission [ml]	*p* = 0.004	OR = 1.006	95% CI: 1.002–1.010
Infarct volume at admission [ml]	*p* < 0.001	OR = 1.015	95% CI: 1.007–1.022
History of cancer	*p* = 0.645	OR = 2.456	95% CI: 0.974–6.365
ICA occlusion	*p* = 0.705	OR = 1.204	95% CI: 0.460–3.147
T-occlusion	*p* = 0.919	OR = 1.082	95% CI: 0.238–4.918
M1 occlusion	*p* = 0.370	OR = 1.441	95% CI: 0.647–3.212
Successful recanalization	*p* < 0.001	OR = 0.240	95% CI: 0.117–0.497

*HMCAS* hyperdense middle cerebral artery sign, *ICA* internal carotid artery

## Discussion

Our study is, to the best of our knowledge, first to investigate the relationship between the presence of HMCAS and one-year functional outcome and mortality of anterior circulation AIS patients treated with MT. Our results show that HMCAS is independently associated with both one-year functional outcome and mortality, as well as lower three months mortality but not three months functional outcome.

In our study group HMCAS was present in the majority of MT-treated anterior circulation AIS patients (67.97%), which is similar to the results of other Authors. In a recent meta-analysis, the occurrence of HMCAS was 62.15% [23] and it generally ranges from about 47% [10] to 73% [8].

We have found higher prevalence of patients with recent malignancy history in non-HMCAS group. According to literature reports patients with cancer-related stroke (defined by other Authors as having AIS with active cancer with no other possible causes of stroke, except for hypercoagulability [24, 25]) often have thrombi enriched in platelets and fibrin, and relatively fewer erythrocytes [24, 26]. It has been suggested that such clots may exhibit lower density on NCCT [27, 29]. Although this raises the possibility that thrombus composition could contribute to the lower frequency of HMCAS in patients with malignancy, this remains speculative. Importantly, the presence of a recent malignancy in our cohort does not necessarily imply a cancer-related stroke, as alternative aetiologies are also possible.

On the other hand, the erythrocyte-rich clots, that are more commonly hyperdense according to several studies [27–29], have been proven to have increased viscosity and decreased elasticity [30]. There are reports that HMCAS clots respond better to mechanical thrombectomy [8, 31], however many authors note only non-significant difference in favour of HMCAS [9, 12, 14, 15]. Our results show also only non-significant difference in successful recanalization rate in favour of HMCAS group. It may be speculated that histological differences between the HMCAS (+) and HMCAS (−) groups could also contribute to differences in clot permeability and, consequently, to the variation in baseline penumbra volume that we observed. Such differences might also influence clot mobility, resulting in variations in occlusion location. However, these hypotheses could not be confirmed in the present study due to the lack of pathological examination of thrombi and should be evaluated in future research.

The reason for more common IVT treatment in HMCAS (+) group is difficult to explain. The difference might be related to the fact that high specificity of HMCAS confirm ischaemic aetiology of the symptoms and encourages fast causative treatment in the referring centres (in patients transported in drip and ship model) before referring to the Comprehensive Stroke Center for further evaluation and mechanical thrombectomy.

Some authors report that non-HMCAS occlusions are more often caused by in-situ thrombosis due to local atherosclerosis [13, 14], however other research [8, 9] do not find such relationship. Our study has found similar prevalence of large vessel disease in HMCAS (+) and (-) groups. The relationship between stroke aetiologies and thrombus density should be further studied, and our data might be useful for further meta-analyses addressing this issue.

It is reported in literature that HMCAS in patients not receiving reperfusion treatments is related to poor functional outcome, both in research from pre-thrombectomy era [32] and in modern studies involving patients with large infarctions [33]. HMCAS is also reported as poor prognostic factor for patients treated only with thrombolytic therapy [34, 37].

There were few published studies investigating the relationship between HMCAS and outcome in AIS patients who undergo MT. Most of the studies did not find HMCAS association with functional outcomes or mortality [9, 11–14]. Some of those studies involved only patients with MCA occlusion, others with ICA as well. Recent meta-analysis including 724 patients also did not find significant influence of HMCAS on functional outcome at 90 days and mortality but there is a visible tendency towards better outcomes in newer studies [23]. One recent study on a sample of patients with M1, M2 and tandem occlusion showed an association between HMCAS and three month good functional outcome (OR 2.48, 95% CI: 1.10–5.58, *p* = 0.028) and in-hospital mortality (OR 0.29, 95% CI: 0.1–0.81, *p* = 0.018) but not with three month mortality (OR 0.48, 95% CI: 0.19–1.23, *p* = 0.126) [8]. In another one concerning only M1 occlusions it was shown that absence of HMCAS was correlated with worse functional outcome (OR 2.83, 95% CI 1.29–6.17, *p* = 0.009) and higher mortality (48.4% vs. 22.5% in HMCAS group, *p* = 0,009) after three months. Authors hypothesised that exclusion of M2 patients was important in achieving this result, as their prognosis is better [15]. One recent study found a negative correlation between the presence of HMCAS and good functional outcome in patients with MCA occlusion with cardioembolic etiology treated with MT (OR 0.193 (0.040–0.937), *p* = 0.041), but not in patients with large vessels disease etiology [36]. Another one showed that in the group of patients with M1 occlusion, HMCAS patients more commonly achieve functional independence (mRS 0–2) after direct thrombectomy, but this was not the case in patients who received IVT as bridging therapy [38].

The difference in 365-day versus 90-day functional outcomes may be due to a better response to long-term rehabilitation response in HMCAS (+) patients. Although HMCAS is typically linked to more severe strokes, its presence may have enabled faster diagnosis and more effective acute treatment. These early advantages might not have impacted short-term outcomes but could have contributed to improved recovery over time. This difference in outcomes warrants further investigation in studies designed to gather more information on the long-term rehabilitation and treatment process, while also incorporating radiological markers in acute stroke.

In one study Authors reported lower prevalence of ICH (OR 0.49, 95% CI 0.25–0.97, *p* = 0.042) and symptomatic ICH (SICH; OR 0.16, 95% CI 0.04–0.63, *p* = 0.009) in HMCAS (+) group. They also showed that the better outcomes in HMCAS (+) patients can be mediated via lower prevalence of SICH [8]. Another research, focusing on haemorrhagic transformation, showed relationship between presence of proximal hyperdense clot and prevalence of asymptomatic intracranial haemorrhage, compared to proximal isodense clot (OR 2.27 (1.29–3.99), *p* = 0.004) [10]. In another study, HMCAS was associated with more frequent ICH after MT (OR 8.55 (3.01–24.31), *p* < 0.001) [35]. Recent meta-analysis showed no significant effect of HMCAS on occurrence of SICH [23]. Our data did not show impact of HMCAS on ICH occurrence which is consistent with most of the published studies [11–13, 36].

There are several limitations in this study. It is a single centre observation, which limits the sample size and diversity of population. The sample size has been further reduced by missing basal CT images or loss of follow-up of some patients. However, number of analysed patients is still higher than in the other original research concerning influence of HMCAS in patients undergoing MT. More than half of the patients were admitted in a drip and ship model, meaning that they were originally admitted to regional hospitals and then transferred to our reference unit. Therefore, their original imaging data were acquired according to different protocols and using different equipment, and this research cannot draw any conclusions concerning e.g. slice thickness in HMCAS assessment. Furthermore, we did not collect premorbid mRS score which serve as indicator of baseline functional status and make follow-up evaluation of this status more objective. Its absence may have led to overestimation of poor outcomes in patients with pre-existing disability. While this limitation does not affect internal comparisons between groups in our cohort, it does restrict the generalizability of our findings. Moreover, in the group comparison we observed heterogeneity, as indicated by a statistically significant difference (*p* < 0.05), with respect to several variables discussed above. However, we showed that HMCAS presence is independently associated with positive outcomes despite these differences. Another limitation is that the study design did not allow us to explain the difference between the 90-day and 365-day functional outcomes, underscoring the need for further research into the potential mechanisms involved. We did not include stratified analyses by HMCAS location in this manuscript in order to maintain clarity of the narrative. This omission may have reduced the study’s ability to verify certain pathophysiological hypotheses; such subgroup analyses should be addressed in future studies with a more focused aim.

In conclusion, our study provides real-world evidence that the presence of a hyperdense middle cerebral artery sign on admission NCCT is associated with favourable long-term outcomes in AIS patients undergoing MT. The originality of our work lies in the extended one-year follow-up, which add to the limited evidence from previous studies with shorter observation periods. These findings suggest that HMCAS may serve as a useful prognostic marker in clinical practice. Nevertheless, multicentre studies with larger cohorts are needed to confirm our results and to further explore the underlying mechanisms.

## Data Availability

No datasets were generated or analysed during the current study.
